# Supplementary material for: Quantifying Human Engagement into Playful Activities
Source: Sci Rep. 2020 Mar 5;10:4145. doi: 10.1038/s41598-020-60742-8 (PMC7058085; doi:10.1038/s41598-020-60742-8)
Supplement: Supplementary file 1 — Supplementary Information. [file 41598_2020_60742_MOESM1_ESM.pdf]

Supplementary Information for: Quantifying Human Engagement into Playful Activities

David Reguera[1,2], Pol Colomer de Simón[1,2], Iván Encinas[3], Manel Sort[3], Jan Wedekind[3], and Marian Boguñá[1,2]

[1]Departament de Física de la Matèria Condensada, Facultat de Física, Universitat de Barcelona, Martí i Franquès 1, 08028 Barcelona, Spain
[2]Universitat de Barcelona Institute of Complex Systems (UBICS), Universitat de Barcelona, 08028 Barcelona, Spain
[3]King Digital Entertainment, 08029 Barcelona, Spain

1 A Continuous Time Random Walk (CTRW) Model of Player Progression and Retention

Suppose we have a simple linear game where players can access the different levels one by one. The main goal of this model is to evaluate the survival probability of the game $S(t)$, that is, the probability that a given player keeps playing the game after some time t, counted from the time the player started playing the game for the first time. In our approach, time is treated as continuous, players are considered as identical and independent, and always progress forward in an increasing manner[1]. In addition, the assumptions that we make are as follows:

1. When a player reaches a new level n, it takes him/her a random time to pass it. This time is controlled by the probability density function (pdf) $\psi_n^p(t)$ that, in general, will depend on the particular level n.
2. On the other hand, being at level n, the player can get bored or frustrated and abandon the game after another random time that follows the pdf $\psi_n^a(t)$, also dependent on the level n.
3. To simplify the model, we assume that these two random times are statistically independent. This means that in order to pass a level, the random time given by the pdf ψ_n^p has to be smaller than the time given by ψ_n^a.

The main quantity of interest is the probability that the player is at level n at time t, $P_n(t)$. The survival probability of the game can be computed from this distribution as

$$S(t) = \sum_{n=1}^{\infty} P_n(t). \tag{S1}$$

The probability $P_n(t)$ satisfies the following equation [2]

$$P_n(t) = \int_0^t h_n(\tau)\Psi_n^p(t-\tau)\Psi_n^a(t-\tau)d\tau, \quad \text{for } \; n \geq 1 \tag{S2}$$

where $\Psi_n^p(t)$ and $\Psi_n^a(t)$ are the corresponding survival probabilities, that is $\Psi_n^p(t) = \int_t^\infty \psi_n^p(\tau)d\tau$ and $\Psi_n^a(t) = \int_t^\infty \psi_n^a(\tau)d\tau$, representing the probability that the time required to pass or abandon, respectively at level n is larger than t. In turn, $h_n(t)$ is the probability that the player has reached level n between t and $t+dt$, with the initial condition $h_1(t) = \delta(t)$. Eq. (S2) thus represents the probability that a jump was made to level n at time $\tau \leq t$ and no further transitions to the next level or abandons took place. The function $h_n(t)$ satisfies the following self-consistent equation

$$h_n(t) = \int_0^t h_{n-1}(\tau)\psi_{n-1}^p(t-\tau)\Psi_{n-1}^a(t-\tau)d\tau, \quad \text{for } \; n \geq 2. \tag{S3}$$

Notice that the integrals in the last two equations are convolutions, meaning that they can be solved using Laplace transforms. Denoting by $\hat{h}_n(s)$ the Laplace transform of function $h_n(t)$, we can solve it as

$$\hat{h}_n(s) = \prod_{i=1}^{n-1} \mathcal{L}\left\{\psi_i^p\Psi_i^a\right\}(s) \quad \text{for } \; n \geq 2 \tag{S4}$$

where $\mathcal{L}\left\{\psi_i^p\Psi_i^a\right\}(s)$ denotes the Laplace transform of the product of functions $\psi_i^p(t)$ and $\Psi_i^a(t)$. Using this expression, we can finally write a general formula for the Laplace transform of the survival probability

$$\hat{S}(s) = \mathcal{L}\left\{\Psi_1^p\Psi_1^a\right\}(s) + \sum_{n=2}^{\infty} \mathcal{L}\left\{\Psi_n^p\Psi_n^a\right\}(s) \prod_{i=1}^{n-1} \mathcal{L}\left\{\psi_i^p\Psi_i^a\right\}(s). \tag{S5}$$

It is quite easy to check the consistency of this expression by considering the case when the player never abandon the game and so $\Psi_n^a(t) = 1 \; \forall n$. In such case, the Laplace transform $\hat{S}(s) = 1/s$ and, thus, $S(t) = 1$.

To make further progress, we need to make some assumptions about the particular form of the probability density functions at each level. We first consider a non-homogeneous Poisson distribution for the abandon time, that is,

$$\Psi_n^a(t) = e^{-k_a(n)t} \tag{S6}$$

where $k_a(n)$ is the abandon rate, that in general depends on the particular level n. Thanks to the properties of the Laplace transform, in this case, the Laplace transform of the product of functions that appears in Eq. (S5) is just the Laplace transform of the distributions $\psi_n^p(t)$ but with the argument shifted by a factor $k_a(n)$. Using this property and after some algebra, we can write

$$\hat{S}(s) = \frac{1}{s+k_a(1)} + \sum_{n=1}^{\infty} \frac{k_a(n) - k_a(n+1)}{[s+k_a(n)][s+k_a(n+1)]} \prod_{i=1}^{n} \hat{\psi}_i^p(s+k_a(i)). \tag{S7}$$

Notice that if the abandon rates are independent of the levels, then $k_a(n) = k_a$ and the survival probability is just $S(t) = e^{-k_a t}$, independently of the distributions $\psi_n^p(t)$. This is easy to understand as in this case the abandon process is a simple homogeneous Poisson process and, thus, independent of the particular levels the player has achieved. Equation (S7) is also interesting because it tells us that in order to have a non trivial result, it is necessary that there is a dependence of the abandon

rate on the different levels. The equation is also interesting because by setting $s = 0$, we obtain a closed formula for the average survival time $\bar{t}$, which reads

$$\bar{t} = \frac{1}{k_a(1)} + \sum_{n=1}^{\infty} \frac{k_a(n) - k_a(n+1)}{k_a(n) k_a(n+1)} \prod_{i=1}^{n} \hat{\psi}_i^p(k_a(i)) \tag{S8}$$

which gives us the contribution of each individual level to the overall average survival time of the game. An interesting property made evident by Eq. (S8) is that flat levels do not contribute to the average survival time. By flat levels we mean sequences of levels with constant abandon rates and so $k_a(n+1) \approx k_a(n)$. That is, a long sequence of similar levels will never increase the average lifespan of players in the game.

2 Independence of the abandon and pass times

The previous CTRW model for player progression and churn relies on two main inputs: the probability density distributions of the pass and abandon times, $\psi_n^p(t)$ and $\psi_n^a(t)$. For convenience and simplicity, we have assumed that both the pass and abandon times are exponentially distributed, i.e.

$$\psi_n^p(t) = \frac{1}{\bar{t_p}(n)} e^{-t/\bar{t_p}(n)} \tag{S9}$$

and

$$\psi_n^a(t) = \frac{1}{\bar{t_a}(n)} e^{-t/\bar{t_a}(n)} \tag{S10}$$

where $\bar{t_p}(n)$ and $\bar{t_a}(n)$ are the average time to pass or abandon at level n, respectively. In this case, the average times to pass or abandon at level n are just the inverse of the pass and abandon rates, specifically

$$k_p(n) = 1/\bar{t_p}(n) \tag{S11}$$

$$k_a(n) = 1/\bar{t_a}(n). \tag{S12}$$

The main parameters of the model, namely the average times to pass, $\bar{t_p}(n)$, or abandon, $\bar{t_a}(n)$, each level n, can be directly measured from the datasets in terms of the probability to churn at level n, $p_c(n)$, and the empirical time to pass level n, $\bar{t_p}^{emp}$, as explained in the methods section. Quite interestingly, these empirical measures provide a strong empirical evidence in favor of our model. We first notice that $\bar{t_p}^{emp}$ and $p_c(n)$ are independent empirical measures. As such, one could have chosen to model the evolution of this process starting directly with these two functions. However, as we show below, both measures are strongly correlated. Interestingly, our CTRW model provides a natural explanation for such correlations. The probability to churn at a given level $p_c(n)$ is typically small, implying that in general we can approximate Eq. (2) as

$$p_c(n) \approx \frac{\bar{t_p}(n)}{\bar{t_a}(n)} \text{ and } \bar{t_p}^{emp} \approx \bar{t_p}(n) \tag{S13}$$

and, therefore, $p_c(n)$ and $\bar{t_p}^{emp}$ should be positively correlated. Figure S1 shows such correlations for the Candy Crush Saga dataset. On the other hand, abandon and pass times are assumed to be independent. The validity of this assumption can be tested by analyzing the correlation between both times in the dataset. Fig. S2 shows the results of a detrended fluctuation analysis of the data in Fig. 2, demonstrating that abandon and pass times are indeed truly uncorrelated.

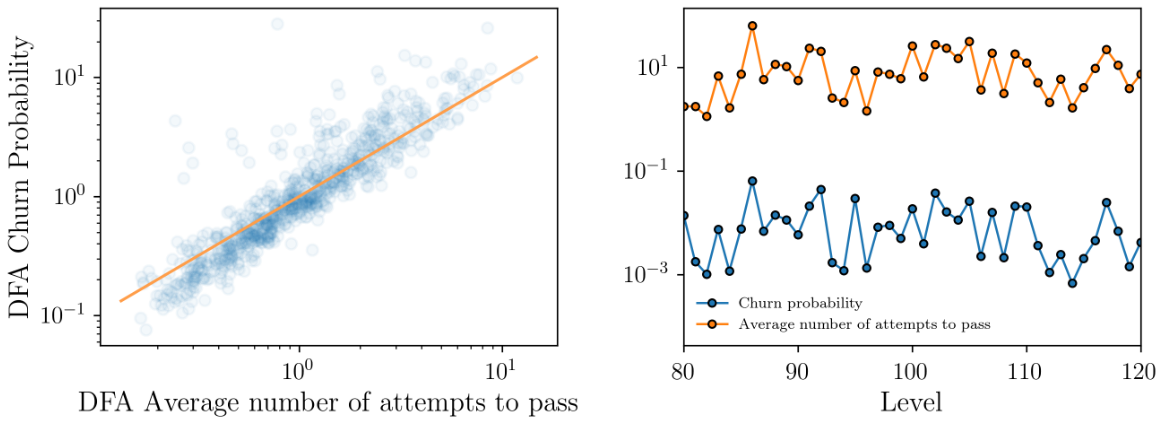

Figure S1: Left: Scatter plot showing the detrended correlation between churn probabilities and the average number of gameplays required to pass a particular level for the Candy Crush Saga dataset shown in Fig. 2 of the main text. The orange line indicates the direct proportionality with slope 1. Right: Snapshot of churn probabilities and average number of gameplays to pass a given level as a function of the level. The similarities in both lines provide a clear evidence of the correlation between them.

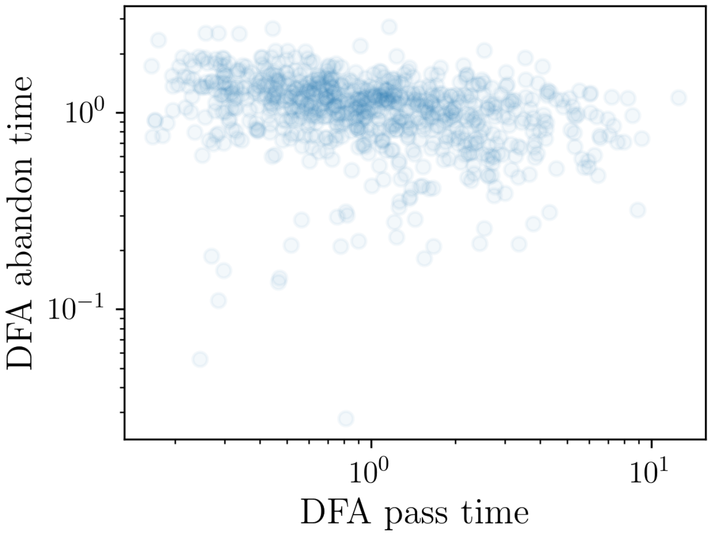

Figure S2: A detrended fluctuation analysis of the data in Fig. 2, indeed demonstrates that abandon and pass times are truly uncorrelated.

3 Verification of the exponential behavior of the abandon and pass time distributions

From the dataset, one cannot measure directly the probability distribution function of abandon, $\psi_n^a(t)$, and pass times, $\psi_n^p(t)$ of a specific level. This is due to the fact that abandon and pass times are unconditioned random processes. That is, $\psi_n^p(t)$ accounts for the distribution of pass times at level n if players were not allowed to quit the game, which is a condition that is not meet in a real dataset. Similarly, $\psi_n^a(t)$ is the distribution of abandon times at level n if players were not allowed to quit the game. Instead, the distributions that we can observe directly are the *empiric* distribution of pass and abandon times. These distributions can be simply obtained as the normalized histogram of the attempts required to pass or abandon a specific level, and are mathematically given by

$$\bar{\psi}_n^p(t) = \frac{\psi_n^p(t)\,\Psi_n^a(t)}{\int_0^\infty \psi_n^p(t)\,\Psi_n^a(t)} \tag{S14}$$

$$\bar{\psi}_n^a(t) = \frac{\psi_n^a(t)\,\Psi_n^p(t)}{\int_0^\infty \psi_n^a(t)\,\Psi_n^p(t)} \tag{S15}$$

The right hand side of the previous equations represents the distribution of pass times conditioned to the fact that the player has not yet churned, and the distribution of abandon times conditioned to the fact that the player has not yet passed level n at time t, respectively. In the case that the unconditional probabilities are exponentially distributed as in Eqs. (S9) and (S10), it is easy to show that the Complementary Cumulative Distribution Function (CCDF) of the empiric distributions of pass and abandon times is just given by

$$\bar{\Psi}_n^p(t) = \bar{\Psi}_n^a(t) = e^{-t/\bar{t}^{emp}(n)} \tag{S16}$$

where $\bar{t}^{emp}(n) = \bar{t}_p(n) + \bar{t}_a(n)$. Fig. S3 represents the CCDF for different levels of Blossom Blast Saga, plotted as a function of the number of attempts divided by the corresponding empiric mean time.

The CCDF distribution for all levels nicely collapses in a single master curve that is very well approximated by the predicted exponential behavior, demonstrating that, to a good approximation, the distribution of abandon and pass times are indeed exponential.

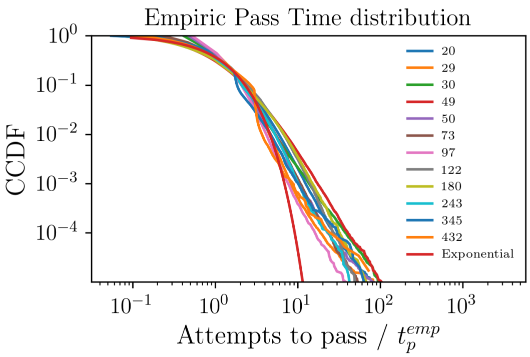

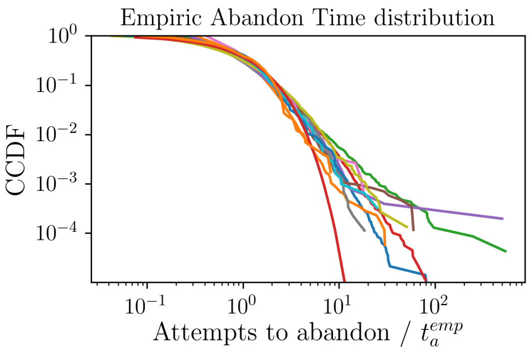

Figure S3: Complementary Cumulative Distribution Function (CCDF) of the pass (left) and abandon (right) times, for randomly selected levels of the Blossom Blast Saga game, plotted as a function of the number of attempts divided by the corresponding empiric pass and abandon time. The red line represents in both cases, the expected behavior if the abandon and pass times are exponentially distributed. Data corresponds to a cohort of 4,568,124 players with install dates from 1-1-2016 to 31-1-2016 in all platforms, followed for two years.

4 “Universality” of the power-law dependence of the abandon times

We have measured from different datasets the abandon and pass times of each individual level for players of different continents, playing using a different platform, and that have installed the game and are playing at different periods of time intervals. In all cases, for each game we obtained almost identical average pass times (not shown) and a consistent power-law behavior of the average abandon times with very similar exponents (see Fig. S4). This is a clear indication of the “universal” power-law behavior of the engagement in this fun activity.

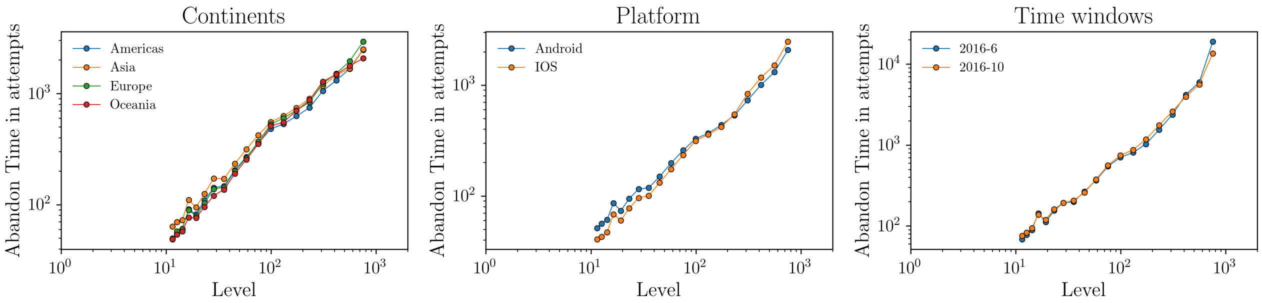

Figure S4: Average abandon times measured at each level of the game Candy Crush Soda Saga from all players in the period 1-06-2016 to 31-07-2016. The data has been binned and plotted in double logarithmic scale. Left: Abandon time measured for players segmented according to their continent. Middle: Abandon time measured for players using Android or OS as platform. Right: Abandon time measured for different time periods, corresponding to June and October 2016. In all cases, a clear power-law behavior is observed.

5 Finite size effects

The estimation of average abandon and pass times are affected by the length of the dataset, that is, the time span during which we follow our cohort of players. This is so because empirically we consider that a player has abandoned the game at his/her last observed gameplay. However, if we increase the observation time window, some players may be still active in the game even thought they were considered as non-active with the smallest time window. This affects the estimation of $p_c(n)$ and, thus, of $\bar{t_p}(n)$ and $\bar{t_a}(n)$. These effects are more evident in Fig. S5 comparing how the number of alive players after a given number of gameplays or levels, and the abandon and pass times change with the period of time used in their evaluation. Whereas the pass times seem to be quite stable, the tail of the abandon times is strongly affected by data-censorship due to the finite time window of analysis. However, as the time window increases, we observe a clear collapse towards a clean power law behavior.

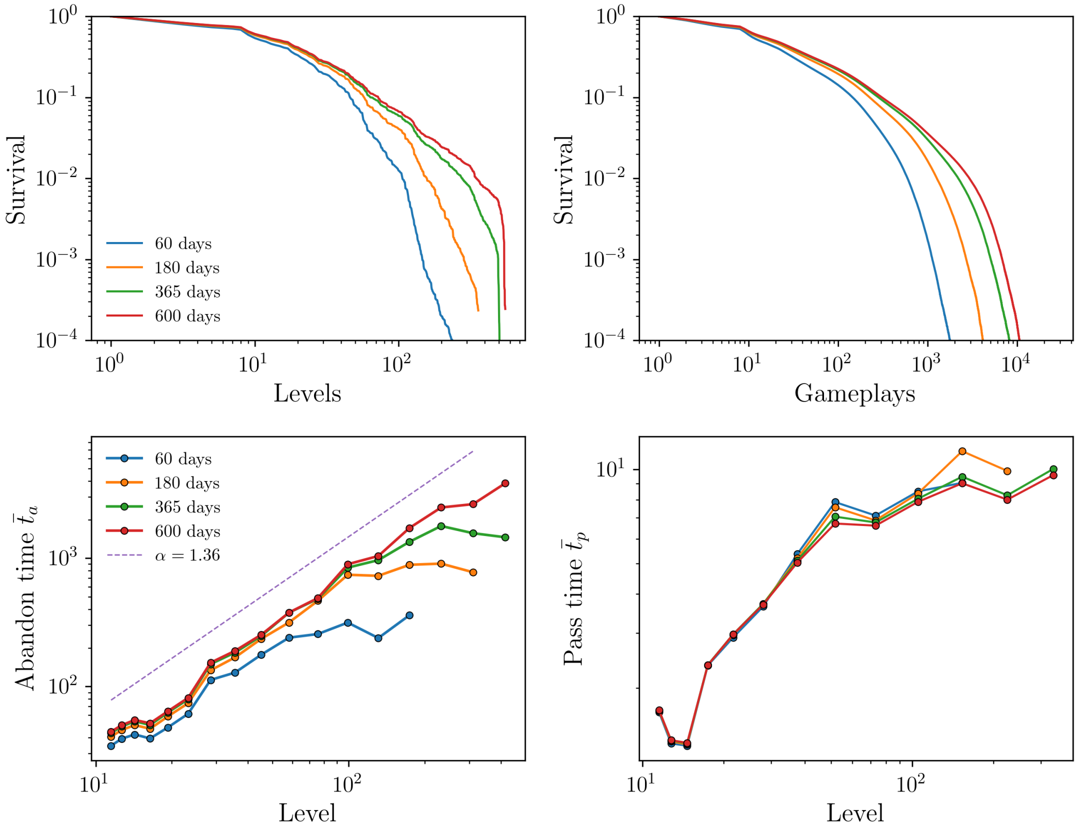

Figure S5: Illustration of the finite time window effects. The plots represent the change in the fraction of players still active in the game after a total number of gameplays (a) or levels (b), the mean abandon (c) and pass times (d), for Papa Pear Saga dataset on the Facebook interface of a week cohort of users with installation date from 11/10/2013 to 18/10/2013 measured using different intervals of real time activity, spanning from 2 to 24 months. For short time windows, most players have not had the time to play high levels and this leads to a clear cut-off in the survival curves and a plateau in the observed abandon times.

6 Phase transition

The model undergoes a phase transition between a phase where all players eventually quit the game and a phase where a finite fraction of players never abandon the game. The probability of a player to be still playing at level n is simply the probability of not having churned in any level below n, that is,

$$S(n) = \prod_{i=1}^{n} (1 - p_c(i)). \tag{S17}$$

For all levels, $p_c(n)$ is always a small number and, therefore, we can approximate this expression as

$$S(n) \approx e^{-\sum_{i=1}^{n} p_c(i)} \approx e^{-\int_{i=1}^{n} p_c(i) di} \tag{S18}$$

where in the last approximation we have taken the continuum approximation. Using Eq. (S13) and assuming that $\bar{t}_a(n) = an^\alpha$ and $\bar{t}_p(n) = bn^\beta$, $S(n)$ can be expressed as

$$S(n) \approx e^{-\frac{b}{a}\int_1^n i^{\beta-\alpha} di} = \begin{cases} e^{-\frac{b}{a(\beta-\alpha+1)}(n^{\beta-\alpha+1}-1)} & \alpha \neq 1+\beta \\ \\ \frac{1}{n^{b/a}} & \alpha = 1+\beta \end{cases} . \tag{S19}$$

When $\alpha < 1+\beta$, the limit $\lim_{n\to\infty} S(n) = 0$, which implies that all players eventually abandon the game. However, when $\alpha > \alpha_c = 1+\beta$, the survival probability $S(n)$ converges to a constant value. Therefore, in this case, there is finite probability that a player never abandon the game S_∞ given by

$$S_\infty = e^{-\frac{b}{a(\alpha-\alpha_c)}}. \tag{S20}$$

Notice that S_∞ and all its derivatives of any order vanishes at $\alpha = \alpha_c$ (evaluated from the right) so that the phase transition is of infinite order. When $\alpha > \alpha_c = 1+\beta$, the survival probability of players with finite lifespan can be evaluated as

$$S_{fin}(n) = \frac{S(n) - S_\infty}{1 - S_\infty}, \tag{S21}$$

that, for $n \gg 1$ behaves as $S_{fin}(n) \sim n^{\alpha_c-\alpha}$.

The interpretation is then as follows: for $\alpha \ll \alpha_c$ players' lifespans are short. When $\alpha \approx \alpha_c$ from below, the average lifespan grows and diverges right at the critical point, even though all players eventually abandon the game. Above the critical point $\alpha > \alpha_c$, there is a fraction of players that never abandon the game, and those that do abandon the game follow a power law distribution with exponent $\alpha - \alpha_c + 1$. When $\alpha \gg \alpha_c$, players either stay in the game forever or have a very short lifespan, abandoning the game at very low levels.

References

[1] In real games players that have reached a given maximum level sometimes chose to play lower levels. These events are not very common (e.g. in Papa Pear Saga, less than 2% of gameplays are played at lower levels) and our model ignores them. The process that we model correspond to the stochastic evolution of the maximum level achieved by the player.

[2] E. W. Montroll and G. H. Weiss, J. Math. Phys. 6, 167 (1965).

[3] See for instance: R. Erban, J. Chapman and P.K. Maini, "A practical guide to stochastic simulations of reaction-diffusion processes", Lecture Notes, available as http://arxiv.org/abs/0704.1908, 35 pages (2007).
